# Bright Light Therapy for Parkinson Disease: A Literature Review and Meta-Analysis of Randomized Controlled Trials

**DOI:** 10.3390/biology10111205

**Published:** 2021-11-19

**Authors:** Hsu-Tung Huang, Tsai-Wei Huang, Chien-Tai Hong

**Affiliations:** 1 Department of Neurology, Shuang-Ho Hospital, Taipei Medical University, New Taipei City 23561, Taiwan; ts02229378@yahoo.com.tw; 2 Cochrane Taiwan, Taipei Medical University, Taipei 11031, Taiwan; tsaiwei@tmu.edu.tw; 3 School of Nursing, College of Nursing, Taipei Medical University, Taipei 11031, Taiwan; 4 Center for Nursing and Healthcare Research in Clinical Practice Application, Wan Fang Hospital, Taipei Medical University, Taipei 116081, Taiwan; 5 Department of Neurology, School of Medicine, College of Medicine, Taipei Medical University, Taipei 11031, Taiwan

**Keywords:** Parkinson disease, bright light therapy, meta-analysis

## Abstract

**Simple Summary:**

Parkinson’s disease (PD) is a common neurodegenerative disease that manifests as motor dysfunction and nonmotor symptoms (NMSs). Apart from motor symptoms, NMSs include sleep disorders, neuropsychiatric problems, and cognitive impairment, which negatively influence patients’ daily lives and caregivers. Disturbances of the sleep cycle also worsen overall health, causing dysregulation of cortisol and melatonin secretion. Furthermore, bright light therapy (BLT) is a well-known treatment for circadian rhythm sleep disorders, seasonal affective disorders, and dementia-related sleep disturbances under the regulation of circadian rhythm by melatonin, a chronological pacemaker. BLT is also applied to treat depressive symptoms and bipolar disorder through unknown mechanisms. The present study, at first, conducted a literature review, which found that a few non-controlled studies demonstrated improvements in motor symptoms and NMSs in PD. Secondly, the present study performed a meta-analysis of the randomized controlled trials which treated the PD patients with BLT. The results revealed that BLT nonsignificantly alleviated symptoms of depression and sleep disorders in patients with PD. However, the inconsistency between BLT protocols, such as varied timing, dosages, and treatment durations, may render BLT’s efficacy challenging to demonstrate, and future RCTs must be obtained.

**Abstract:**

Sleep disorders and depression are significant nonmotor symptoms (NMSs) of Parkinson disease (PD). However, few effective, evidence-proven medical treatments are available for alleviating these symptoms. Bright light therapy (BLT) is a well-established treatment for circadian rhythm sleep disorders and seasonal affective disorder. The present study conducted a literature review for the effect of BLT on PD, especially a meta-analysis of randomized controlled trials (RCTs). We searched for studies using the PubMed and Cochrane Library databases. The major outcomes were the effects on sleep and depression. The effect on motor symptoms was also analyzed as a secondary outcome. This study was registered with PROSPERO (CRD42020204454). Six studies were included in the literature review only, and the other five RCTs were included in the meta-analysis. Despite the positive effects of BLT on PD patients, which were demonstrated in noncontrolled studies, in the meta-analysis of the RCTs, BLT did not significantly improve the depressive symptoms (standardized mean difference (SMD): −0.15, 95% confidence interval (CI): −0.48 to 0.17, *p* = 0.36) and excessive daytime sleepiness (EDS) (SMD: −0.12, 95% CI: −0.49 to 0.25, *p* = 0.53) in PD patients. Regarding motor symptoms, no significant beneficial effects were conferred (SMD: −0.11, 95% CI: −0.44 to 0.21, *p* = 0.49). In conclusion, BLT did not significantly alleviate depression and sleepiness. The inconsistency between BLT protocols, such as the varied timing, dosages, and treatment durations, may render BLT’s efficacy difficult to demonstrate. The small effect size obtained from the present meta-analysis indicates that future RCTs are necessary, for which BLT protocols are standardized and more patients are enrolled to determine whether a significant therapeutic benefit was conferred.

## 1. Introduction

Parkinson disease (PD) is a chronic, progressive neurodegenerative disease that manifests as motor dysfunction and nonmotor symptoms (NMSs). The motor symptoms of PD are mainly attributed to the loss of striatal dopaminergic neurons in the midbrain substantia nigra, and the presence of some NMSs, such as dysautonomia, dementia, and depression, indicates the loss of nondopaminergic neurons [1]. The motor symptoms include resting tremor, bradykinesia, muscular rigidity, and postural instability. The NMSs of PD include sleep disorders, neuropsychiatric problems, and cognitive impairment, which may present before the onset of motor symptoms [2]. Neuropsychiatric symptoms of PD, especially depression, have detrimental consequences for the quality of life and daily function, and they are associated with an increased burden of care [3].

Circadian rhythm is the 24-h internal clock that regulates cycles of alertness and sleepiness by responding to light changes, which help humans adapt to changes in our environment and anticipate changes in radiation, temperature, and food availability. The human would not optimize energy expenditure and the body’s internal physiology without this endogenous circadian clock—the circadian pacemaker in the suprachiasmatic nucleus of the hypothalamus. In addition, as the body transitions from light to dark, the body sends inputs to the retinohypothalamic pineal pathway. Thus, disturbances in an individual’s sleep cycle can be significantly detrimental to their overall health, including nonrhythmic regulations of core body temperature, cortisol levels, and melatonin secretion [4].

Bright light therapy (BLT) is a well-established treatment for circadian rhythm sleep disorders, seasonal affective disorders, and dementia-related sleep disturbances [5]. The body possesses the circadian clock that determines when humans are in states of sleep and wakefulness. This biological clock and circadian rhythm are regulated by exposure to bright lights, such as sunlight or artificial light [6]. The secretion of melatonin from the pineal gland is inhibited by retinal exposure to light and stimulated in darkness, and the elevation of melatonin triggers physiological sleep. Hence, melatonin is a chronological pacemaker, signaling the environmental light–dark cycle to the organism and the primary control of circadian rhythms [7]. Because of the causal relationship between light exposure and sleep, based on the melatonin-dependent pathway, supplemental exposure to bright light has beneficial effects on sleep quality and daytime vigilance in healthy older people and patients with dementia [8]. Moreover, it has been increasingly applied to treat various sleep and neuropsychiatric conditions [9].

Instead of modulating melatonin expression, BLT is employed to treat depressive symptoms, especially seasonal affective disorder (SAD) and bipolar disorder, through other unknown mechanisms. Several hypotheses have been proposed to explain the effects of light on the mood, including phase shifting of circadian rhythms, alertness enhancement, and improved sleep homeostasis and monoamine neurotransmission [10]. The circadian effect is mediated through phase shifts and the modification of the duration of nocturnal melatonin secretion. Moreover, light stimulates alertness through melatonin suppression, activation of the monoaminergic arousal system, and the simultaneous inhibition of the sleep-inducing ventrolateral preoptic nucleus [11]. Moreover, through the secretion of melanopsin, light directly affects delta sleep activity, as measured using electroencephalograms, and patients with SAD have low delta sleep activity and low sleep efficiency. Finally, light modulates the activation of serotonergic neurons, decreases serotonin reuptake transporter (5-HTT) levels, and increases serotonin (5-HT) levels in mood regulatory areas of the brain [12].

A few studies have examined the effects of supplemental light exposure on patients with PD and documented improvements in symptoms such as depression, bradykinesia, rigidity, dyskinesias, and insomnia [13]. However, some studies have provided negative results; in one study, BLT had no significant beneficial effect on the NMSs of patients with PD [14]. Moreover, most such studies were small-scale open-label studies without proper controls, thus providing a low level of evidence for such effects. Therefore, the present study aimed to summarize the effect of BLT on the NMSs of PD, especially sleepiness and depression, through a meta-analysis.

## 2. Materials and Methods

### 2.1. Inclusion Criteria

This study included clinical trials and observational studies that focused on the therapeutic effect of BLT on PD patients. Only randomized controlled trials (RCTs), which were able to compare the effect of BLT alone on PD with placebo, were further included in the quantitative meta-analysis. RCTs were required to report the patient inclusion and exclusion criteria; the process of randomization; the method, illuminance, and duration of BLT; and a comprehensive assessment of motor symptoms and NMSs, including depression and sleep disorders. This study was registered with PROSPERO (CRD42020204454).

### 2.2. Literature Search Strategy

We searched for literature published before December 2020 in the PubMed, Cochrane Library databases, and Embase following the Preferred Reporting Items for Systematic Review and Meta-Analysis (PRISMA) guidelines. The search keywords are (“bright light therapy” (Title/Abstract) OR “light therapy” (Title/Abstract)) OR “BLT” (Title/Abstract)) AND (“Parkinson’s disease” (Title/Abstract) OR “Parkinson disease” (Title/Abstract)). Only studies published in English were included. All the published studies obtained from the search of these three databases were merged, and the duplications were removed. Afterward, all the remaining studies were screened based on the title and abstract, and the noninterventional (review, viewpoints) and nonclinical (in vivo and in vitro) studies were excluded. Lastly, the remaining studies were assessed through full-text review to determine the allocation: literature review or meta-analysis.

### 2.3. Data Extraction

Baseline and outcome data were independently retrieved by two reviewers (C.-T.H. and H.-T.H.). Furthermore, data on study designs, study population characteristics, and inclusion and exclusion criteria were extracted. Finally, decisions recorded individually by the reviewers were compared, and disagreements were resolved by a third reviewer (T.-W.H.).

### 2.4. Outcomes

Two reviewers (C.-T.H. and H.-T.H.) independently assessed the methodological quality of each study by using the revised risk of bias (version 2.0) method, as recommended by the Cochrane Collaboration. The included studies were scored to determine whether they had a high, medium, or low overall risk of bias. The risk of bias was calculated by assessing five domains: bias resulting from the randomization process, bias resulting from deviations from intended interventions, bias resulting from missing outcome data, bias in the measurement of outcomes, and bias in selecting reported results.

### 2.5. Appraisal of Methodological Quality

The primary outcome was the effect of BLT on depression and excessive daytime sleepiness. The secondary outcome was the changes in PD motor symptoms, assessed using the Unified Parkinson Disease Rating Scale (UPDRS) part III. If more than one depression or sleep quality scale was used in a single study, the more commonly used one was selected for outcome analysis.

### 2.6. Statistical Analysis

Data were entered and analyzed using Review Manager 5.3 (The Cochrane Collaboration, Oxford, UK). A meta-analysis was performed following the PRISMA guidelines. The standard deviation was calculated using the provided confidence interval (CI) limits, standard errors, or interquartile ranges, where appropriate [15,16]. The effect sizes of continuous outcomes were reported as the standardized mean difference (SMD). The precision of effect sizes was reported using a 95% CI. A pooled estimate of weighted mean difference (WMD) was computed using the DerSimonian and Laird random-effects method. A statistically significant result was indicated by *p* < 0.05 or a 95% CI that did not include 1 in the relative risk ratio and 0 in the WMD estimation. Statistical heterogeneity and inconsistency in treatment effects across the studies were evaluated using the Cochrane Q test and I^2^ statistic, respectively. Statistical significance was set at *p* < 0.10 for the Cochrane Q test. Statistical heterogeneity across studies was assessed using the I^2^ statistic, which quantifies the proportion of total outcome variability across studies.

## 3. Results

**Figure 1:**
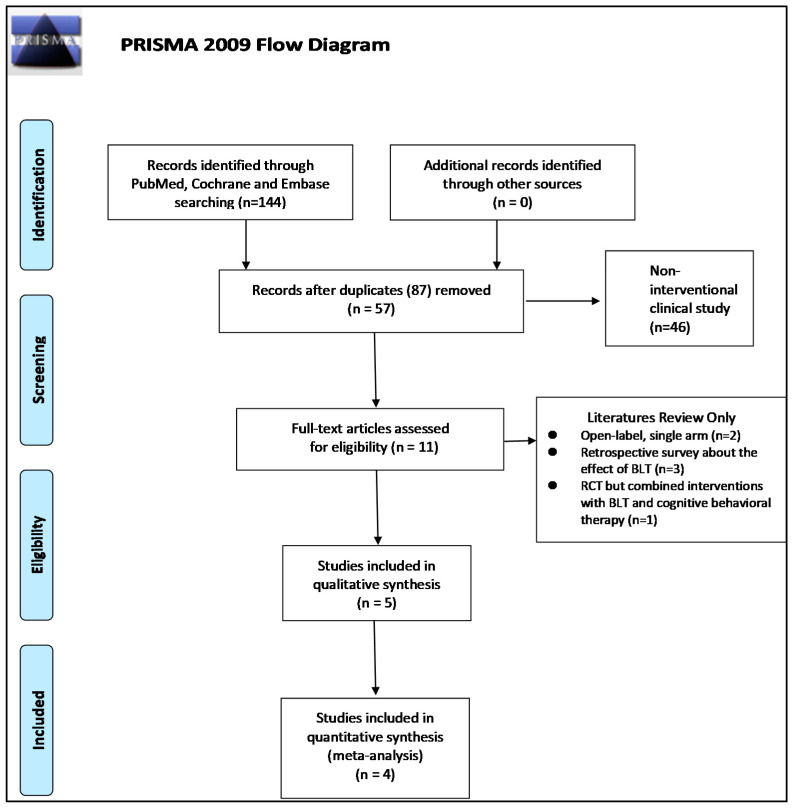
Study selection flowchart. BLT, bright light therapy; RCT, randomized controlled trials.

The flowchart of the screening and selection of the studies is presented in Figure 1. The search was conducted until 31 December 2020. Of 144 publications retrieved in the initial search, 87 were excluded because of duplication. For the remaining 57 studies, 46 studies were excluded by the title and abstract screening because they were not the interventional studies focused on the effect of BLT on PD. Among the remaining 11 studies, 6 of them were RCTs [13,17,18,19,20,21]. However, one study was excluded from the meta-analysis due to the combination of BLT with cognitive behavioral therapy in the interventional group. The rest of the five studies [22,23,24,25,26] were either open-label, single-arm clinical trials or retrospective observational studies focusing on the PD users of BLT. Those six studies excluded from the meta-analysis are summarized in Table 1. They demonstrated a remarkable improvement in motor performance, depression, and sleep quality in PD patients under the BLT treatment. Three studies were conducted from the same institute in Australia, and the rest were from Canada, the Netherlands, and Japan. Willis et al. compared the effect of BLT between PD patients with good compliance and those who quit, and revealed a significant difference in all aspects [26]. Smilowska et al. utilized blue light glasses instead of conventional BLT, which also significantly benefited the sleep parameters [23]. However, substantial heterogeneities were noted, especially the intensity, the daily treatment timing and the duration of each treatment session, and the total duration of BLT treatment (ranging from weeks to years), which was highly discrepant. The detail of the inclusion and exclusion criteria and the light exposure measurement for the qualitative meta-analysis were summarized in the supplementary information (Supplementary Tables S1 and S2).

Regarding the five RCTs in the qualitative meta-analysis, one study was excluded from the quantitative synthesis because it lacked sufficient information on outcomes. Lastly, four eligible RCTs were included in the quantitative meta-analysis. These RCTs included in the qualitative meta-analysis were published between 2007 and 2019 and had sample sizes ranging from 16 to 72. The mean age of patients was approximately 60 years, and most of the study cohorts had male preponderance. All five studies recruited patients diagnosed as having idiopathic PD. Patients in two studies had a stable antiparkinsonian and psychopharmacologic agents regimen for at least four weeks before study screening (Table 2). The risk-of-bias assessments demonstrated a low risk to some concerns in all five studies (Table 3).

**Figure 2:**
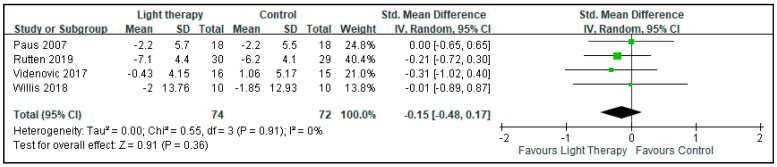
Effect of bright light therapy on the depressive symptoms of patients with Parkinson disease.

**Figure 3:**
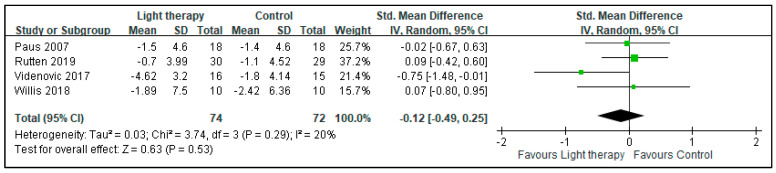
Effect of BLT on the sleepiness of patients with Parkinson disease.

**Figure 4:**
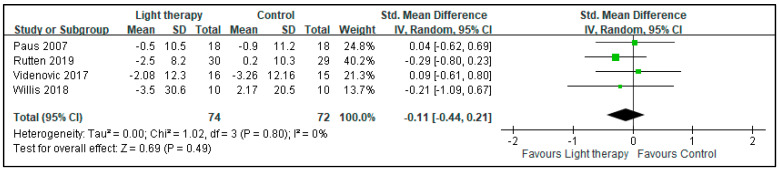
Effect of BLT on the motor symptoms (assessed by Unified Parkinson Disease Rating Scale Part III scores) of patients with Parkinson disease.

The quantitative meta-analysis of depressive symptoms revealed that BLT provided no significant improvements in the symptoms of patients with PD (SMD: −0.15, 95% CI: −0.48 to 0.17, *p* = 0.36). The heterogeneity of this analysis was nonsignificant (I^2^ = 0%; Figure 2). There was no significant beneficial effect on sleepiness (assessed by Epworth Sleepiness Scale in two studies and Daytime Sleepiness subitem from the Scales for Outcomes in Parkinson’s Disease—Sleep in one study) (SMD: −0.12, 95% CI: −0.49 to 0.25, *p*: 0.53) demonstrated. The heterogeneity of this analysis was nonsignificant, with I^2^ = 20% (Figure 3). No beneficial effects were conferred on motor symptoms based on UPDRS-III scores (SMD: −0.11, 95% CI: −0.44 to 0.21, *p*: 0.49). The heterogeneity of this analysis was nonsignificant (I^2^ = 0%; Figure 4).

## 4. Discussion

This study demonstrated that, in the open-label, non-controlled studies, BLT effectively attenuated depressive symptoms and excessive daytime sleepiness in PD patients. However, in the meta-analysis of the RCTs, BLT did not significantly improve those major NMSs in patients with idiopathic PD. We summarized the results of four RCTs published between 2007 and 2019. All four studies recruited patients who were diagnosed as having idiopathic PD by neurologists. Although a possible trend of the therapeutic effects on sleep disorders and depression was noted, the small sample size, even after merging four RCTs, limited our ability to determine whether a statistically significant benefit was conferred.

The management of NMS is challenging in people with PD. Depression is usually treated with conventional antidepressants, as with other non-PD people, despite the considerable adverse effects. Similarly, for the excessive daytime sleepiness (EDS), current evidence only provides “insufficient” evidence for the prescription of modafinil to alleviate EDS in people with PD. Therefore, nonpharmacological treatment is an ideal alternative for them, usually tolerable with minimal adverse effects. In addition, cognitive behavioral therapy and repetitive transcranial magnetic stimulation are possibly effective for PD depression, and continuous positive airway pressure is likely beneficial for the EDS in PD. However, due to the lack of solid evidence, in the latest version of the International Parkinson and Movement Disorder Society Evidence-Based Medicine recommendations on treating PD-NMS, all were recognized as “possibly useful,” and more evidence is required.

BLT is efficacious for the treatment of SAD and nonseasonal depression. However, despite evidence of its efficacy as an antidepressant, the clinical application of BLT remains highly variable internationally [27]. Nevertheless, a hypothesis suggests that BLT may potentially restore sympathovagal balance in patients with major depression disorder [28]. For example, BLT may alleviate SAD by correcting the winter circadian rhythm phase delay or increasing synaptic serotonin [29]. Moreover, in the present meta-analysis, one study published by Sebastian Paus revealed that BLT significantly reduced the Beck Depression Inventory scores of patients with PD; however, the other two RCTs revealed no significant changes in depression, anxiety, or quality of life in patients with PD who received BLT.

BLT is a practical nonpharmacological alternative treatment for sleep disorders, including circadian rhythm sleep disorders, insomnia, and sleep problems related to Alzheimer’s disease and dementia. However, most studies have revealed that only small to medium effect sizes [30] of BLT could be a feasible intervention for impaired sleep–wake cycles associated with PD, especially because BLT has minimal adverse effects. Numerous open-label, single-arm studies have demonstrated the benefit of BLT on PD. Two weeks of white fluorescent light for 1–1.5 h at an intensity of 1000 to 1500 lux once daily, commencing 1 h before the usual time of sleep onset, markedly improved bradykinesia, rigidity, agitation, dyskinesia, mood, sleep, seborrhea, and appetite [22]. Besides, BLT effectively improved sleep in PD patients receiving dopaminergic therapy through a circadian phase shift, indicating a correlation between circadian modulation of BLT and sleep improvement [24]. However, one of the significant limitations of the noncontrolled, open-label study is the possibility of the placebo effect, and the RCTs are required to eliminate it.

Those promising results encouraged researchers to investigate the effect of BLT on PD in the RCT study design. However, the results obtained in the RCTs were not satisfactory. One RCT study indicated that BLT had no remarkable effect on excessive daytime sleepiness [13], whereas another RCT demonstrated that BLT was associated with improvements in excessive daytime sleepiness, sleep fragmentation, sleep quality, and motor symptoms of PD; however, no significant difference was observed between BLT and control groups [19]. In another study, BLT was more effective in improving subjective sleep quality than the control light was, possibly through a BLT-induced decrease in cortisol levels [14]. RCTs published by Aleksandar Videnovic et al. demonstrated an improvement in several sleep parameters according to the Epworth Sleepiness Scale and accounted for from self-reported sleep diaries. The other two studies demonstrated no significant improvement. Perhaps the difference is due to BLT timing, which may cause circadian phase advance or delay.

The present meta-analysis has some advantages and limitations. Although the results were nonsignificant, the meta-analysis method was used to pool all the published RCTs to estimate the possible effect size of BLT on depression and sleep disorders in patients with PD, and this approach could be helpful in further RCTs conducted to determine the effect of BLT on PD or other neurological disorders. However, a significant limitation of this study is the lack of consensus between BLT protocols: the three included studies had different timings, dosages, and treatment durations of BLT, and even different interventions, including lightbox and wearable devices. In addition, there was no objective measurement for comparing different devices. For example, a study published by Paul measured with a luminance meter. Moreover, a study published by Videnovic instructed participants to sit quietly and do other things during therapy to minimize the influence of patient activity and compliance. Second, all included RCTs had a relatively short duration of light exposure. It has been suggested that the duration of BLT exposure must be at least 6 to 8 weeks for BLT’s beneficial effects to emerge. Furthermore, the control group still maintained much daily exposure to ambient bright light during the day, and this exposure was not easy to control [19].

In conclusion, BLT non-significantly alleviated symptoms of depression and sleep disorders in patients with PD. The inconsistency between BLT protocols, such as varied timing, dosages, and treatment durations, may render BLT’s efficacy challenging to demonstrate. The small effect size obtained from the present meta-analysis indicates the necessity of conducting future RCTs with standardized BLT protocols and more enrolled patients to determine whether a significant therapeutic benefit is conferred.

## Figures and Tables

**Table 1:** Characteristics of the included studies for the literature reviews.

Author (Year)	Inclusion Criteria	No. of Patients (Male, %)	Age, Mean (SD or Range)	Intervention	Main Findings	Reason for Not Including into Meta-Analysis
Willis and Turner (2007)	PD with depression or insomnia	12 (67)	66 (46~83)	BLT 1000–1500 lux, 1–1.5 h	Improvement in motor, mood, and sleep	Open-label, single-arm
Willis et al. (2012)	PD	129 (unknown)	66 (12)	BLT 4000–6000 lux, 1–1.5 h	Improvement in motor, mood, and sleep	Retrospective, open-label study
Rios Romenets et al. (2013)	PD with insomnia	BLT + CBT: 6 (100) Placebo: 6 (86)	BLT + CBT: 64.5 (16.3) Placebo: 69.5 (10.5)	BLT 10000 lux, 30 min + weekly CBT	Improvement in sleep and quality of life	Can not clarify the effect of BLT alone
Martino et al. (2018)	PD	140 (65)	66 (10)	BLT 3000–4000 lux, 1–4 h	Improvement in insomnia and RBD	Retrospective, open-label study
Smilowska et al. (2019)	PD	31 (unknown)	unknown	Blue light glasses, 40 lux, usually 30–60 min, twice a day	Improvement in motor, mood, and sleep	Retrospective survey
Endo et al. (2020)	PD	16 (38)	65.4(7.1)	BLT, 5000 lux, 2 h, three months	Improvement in sleep	Open-label, single-arm

PD, Parkinson disease; CBT, cognitive behavioral training; BLT, bright light therapy; RBD, rapid eye movement sleep behavior disorders; SD, standard deviation.

**Table 2:** Characteristics of the included studies suitable for the qualitative meta-analysis.

Author (Year)	Inclusion Criteria	No. of Patients (Male, %)	Age, Mean (SD or Range)	Baseline H and Y Stage	Intervention	Outcome
Paus et al. (2007)	Stage I to IV MMSE ≥ 24	E: 18 (67) C: 18 (61)	E: 63.6 (9.8) C: 63.4 (9.7)	E: 2.7 ± 0.6 C: 2.5 ± 0.4	BLT 7500 lux for 30 min daily for 14 days	BDI, ESS, UPDRS
Videnovic et al. (2017)	Stages I to IV ESS: ≥12	E: 16 (50) C: 15 (33)	E: 62.3 (10.8) C: 64.0 (8.9)	E: 2.1 ± 0.3 C: 2.3 ± 0.5	BLT 10,000 lux for 1 h twice/day for 14 days	BDI, ESS, UPDRS
Willis (2018)	PD	E: 10 (60) C: 10 (50)	E: 66.9 (54–75) C: 66.3 (53–79)	unknown	Polychromatic light, 3000 lux, 1 h for 2 week	BDI, ESS, UPDRS
Rutten et al. (2019)	PD with MDD	E: 35 (57) C: 37 (53)	E: 58.9 (8.5) C: 65.8 (8.6)	E: 2.1 ± 0.6 C: 2.4 ± 0.7	BLT 10,000 lux for 30 min twice/day for 90 days	BDI, ESS, UPDRS, SCOPA-SLEEP
Raymackers et al. (2019)	PD	Cross-over design E-C: 8 (50) C-E:8 (75)	E-C: 66.50 (6.30) C-E: 68.88 (5.89)	unknown	Blue enriched light, average 472.7 lux, 45 min for one month	HADS, ESS

PD, Parkinson disease; MDD, major depressive disorder; MMSE, Mini-Mental State Examination; BLT, bright light therapy; BDI, Beck Depression Inventory; ESS, Epworth Sleepiness Scale; SD, standard deviation, UPDRS, Unified Parkinson Disease Rating Scale; SCOPA-SLEEP, Scales for Outcomes in Parkinson Disease—Sleep Disturbances; HADS, Hospital Anxiety and Depression Scale (HADS).

**Table 3:** Risk-of-bias assessment.

	Allocation Bias	Performance Bias	Attrition Bias	Measurement Bias	Reporting Bias
Paus 2007	Low risk	Low risk	Low risk	Low risk	Low risk
Videnovic 2017	Some concern	Some concern	Low risk	Low risk	Low risk
Willis 2018	Low risk	Low risk	Low risk	Low risk	Low risk
Raymackers 2019	Low risk	Low risk	Low risk	Low risk	Low risk
Rutten 2019	Low risk	Low risk	Some concern	Low risk	Low risk

## Data Availability

All the data was available from the original studies.

## Supplementary Materials

The following are available online at https://www.mdpi.com/article/10.3390/biology10111205/s1, Supplementary Table S1. Characteristics of the Inclusion & Exclusion criteria studies suitable for the qualitative meta-analysis. Supplementary Table S2. Characteristics of the light exposure measurement from the studies suitable for the qualitative meta-analysis.
