# Supplementary material for: Bright Light Therapy for Parkinson Disease: A Literature Review and Meta-Analysis of Randomized Controlled Trials
Source: Biology (Basel). 2021 Nov 19;10(11):1205. doi: 10.3390/biology10111205 (PMC8614737; doi:10.3390/biology10111205)
Supplement: Supplementary file 1 [file biology-10-01205-s001.zip › biology-1280492-supplementary.pdf]

Supplementary Information

Supplementary Table S1. Characteristics of the Inclusion & Exclusion criteria studies suitable for the qualitative meta-analysis.

Author [year]	Inclusion criteria	Exclusion criteria
**Paus et al. [2007]**	Stage I to IV<br>MMSE≥24	If took potentially photosensitizing medication, e.g. imipramine, lithium; changes of any medication during the last two weeks: with suicidal risk in judgment of the investigators; with signs of dementia as measured by MMSE ≤ 23
**Videnovic et al. [2017]**	Stages I to IV; ESS: ≥12 stable PD medication regimen for at least 4 weeks	Atypical parkinsonian syndrome; significant sleep disordered breathing, defined as an apnea-hypopnea index ≥ 15 events per hour; significant periodic limb-movement disorder; REM sleep behavior disorder; cognitive impairment, MMSE ≤ 23, untreated hallucinations or psychosis
**Willis [2018]**	H&Y I to III<br>age ≥ 45<br>receiving dopamine agonists replacement ≥12 months	Concurrent involvement in another study; patients that were medically complicated; history of psychiatric illness; history of alcohol or narcotic abuse; severe depression or suicidal tendencies; pregnancy; use of photosensitizing drugs; pre-existing major joint problems; cognitive impairment or focal neurological deficits.
**Rutten et al [2019]**	PD with MDD according to DSM-IV-TR; stable dose of antiparkinsonian and psychopharmacologic agents ≥4 weeks	Current psychosis; (relative) contraindication for BLT such as a bipolar disorder; increased risk of photosensitization due to medication use or a medical disorder
**Raymackers et al. [2019]**	PD	Participating in other studies were not eligible.

PD, Parkinson disease; MDD, Major Depressive Disorder; MMSE, Mini-Mental State Examination; BLT, Bright light therapy; H&Y, Hoehn and Yahr; ESS, excessive daytime sleepiness; REM, rapid eye movement

Supplementary Table S2. Characteristics of the light exposure measurement from the studies suitable for the qualitative meta-analysis.

Author [year]	Light exposure measurement
**Paus et al. [2007]**	A commercially available white fluorescent light boxes by Philips, model Bright Light Energy, product number HF3304 with head-to-light distance of 20 cm in the active treatment, and 100 cm in the placebo group. Illuminance was measured with a luminance meter and was 7.500 lux in the active treatment and 950 lux in the placebo group.
**Videnovic et al. [2017]**	A light box (SunRay; The SunBox Co) was used for LT administration. The box was placed 86.4 cm away from the subject, and an 86.4-cm string attached from its side.
**Willis [2018]**	A light source containing fluorescent tubes (Apollo BL-6, without ultra-violet emission) with positioning the device at a distance of about 0.8 to 1M from the bridge of the nose to the diffuser.
**Rutten et al [2019]**	A Brazil Lightbox (Lumie, Cambridge, UK), which emits daylight spectrum light with an intensity of 10,000 lux at a 30- to 40-cm distance.
**Raymackers et al. [2019]**	A portable head-mounted device (LuminetteR, Lucimed SA, Villers-le-Bouillet, Belgium) was used for both active BLT and placebo therapy.
